# Genetic Prοpensity for Different Aspects of Dementia Pathology and Cognitive Decline in a Community Elderly Population

**DOI:** 10.3390/ijms26030910

**Published:** 2025-01-22

**Authors:** Stefanos N. Sampatakakis, Niki Mourtzi, Alex Hatzimanolis, Georgios Koutsis, Sokratis Charisis, Iliana Gkelmpesi, Eirini Mamalaki, Eva Ntanasi, Alfredo Ramirez, Mary Yannakoulia, Mary H. Kosmidis, Efthimios Dardiotis, Georgios Hadjigeorgiou, Paraskevi Sakka, Nikolaos Scarmeas

**Affiliations:** 1 1st Department of Neurology, Aiginition Hospital, Athens Medical School, National and Kapodistrian University, 11528 Athens, Greece; stefanos.sab@gmail.com (S.N.S.); nikimourtzi23@gmail.com (N.M.); igelbesi@outlook.com (I.G.); eir.mamalaki@gmail.com (E.M.); e.ntanasi@hotmail.com (E.N.); 2 Department of Psychiatry, Aiginition Hospital, Athens Medical School, National and Kapodistrian University, 11528 Athens, Greece; alhatzi@gmail.com; 3 Neurogenetics Unit, 1st Department of Neurology, Aiginition Hospital, Athens Medical School, National and Kapodistrian University, 11528 Athens, Greece; gkoutsis@med.uoa.gr; 4 Department of Neurology, UT Health San Antonio, San Antonio, TX 78229, USA; scharissis@gmail.com; 5 Division of Neurogenetics and Molecular Psychiatry, Department of Psychiatry and Psychotherapy, Medical Faculty, University of Cologne, 50923 Cologne, Germany; alfredo.ramirez@uk-koeln.de; 6 Department of Neurodegenerative Diseases and Geriatric Psychiatry, University Hospital Bonn, 53127 Bonn, Germany; 7 German Center for Neurodegenerative Diseases (DZNE Bonn), 53127 Bonn, Germany; 8 Department of Psychiatry, Glenn Biggs Institute for Alzheimer’s and Neurodegenerative Diseases, San Antonio, TX 78229, USA; 9 Excellence Cluster on Cellular Stress Responses in Aging-Associated Diseases (CECAD), University of Cologne, 50923 Cologne, Germany; 10 Department of Nutrition and Dietetics, Harokopio University, 17676 Athens, Greece; myianna@hua.gr; 11 Laboratory of Neuropsychology and Behavioral Neuroscience, School of Psychology, Aristotle University of Thessaloniki, 54124 Thessaloniki, Greece; kosmidis@psy.auth.gr; 12 Department of Neurology, University Hospital of Larissa, Faculty of Medicine, School of Health Sciences, University of Thessaly, 41334 Larissa, Greece; edar@med.uth.gr; 13 Department of Neurology, Medical School, University of Cyprus, 1678 Nicosia, Cyprus; g.hadjigeorgiou@euc.ac.cy; 14 Athens Association of Alzheimer’s Disease and Related Disorders, 11636 Marousi, Greece; vsakka@ath.forthnet.gr; 15 Department of Neurology, The Gertrude H. Sergievsky Center, Taub Institute for Research in Alzheimer’s Disease and the Aging Brain, Columbia University, New York, NY 10027, USA

**Keywords:** cognitive decline, amyloid beta, white matter hyperintensities, dementia, pathophysiology, polygenic risk score

## Abstract

In the present study, we investigated the association of genetic predisposition with specific dimensions of dementia pathophysiology for global and domain-specific cognitive decline in older adults. The sample was drawn from the Hellenic Longitudinal Investigation of Aging and Diet (HELIAD) study, comprising 512 cognitively normal individuals over 64 years of age, with a mean follow-up of 2.9 years. Cognitive function was evaluated through a neuropsychological test battery, while genetic predisposition was assessed based on two distinct Polygenic Risk Scores (PRS) for amyloid-beta 42 (Aβ_42_) and white matter hyperintensities (WMH). The association of each PRS with the cognitive decline rate was examined using generalized estimating equation models. In the whole sample, higher PRSs Aβ_42_ (β = −0.042) and WMH (β =−0.029) were associated with a higher rate of global cognitive decline per year, an association which remained significant in age, sex, and education subgroups. Moreover, higher PRSs Aβ_42_ and WMH were related to significant memory decline only in females, older, and highly educated participants. Thus, while the association of both PRSs with global cognitive decline over time was independent of age, sex, or education, the relationship of the specific PRSs with the memory decline rate appeared to vary depending on these factors.

## 1. Introduction

Existing data suggest that approximately 75% of new dementia cases are related to Alzheimer’s disease (AD), which is the most common neurodegenerative disease affecting one-third of individuals over 85 years old [1]. The accumulation of β-amyloid (Aβ) plaques and neurofibrillary tangles has been shown to contribute significantly to the pathophysiology of AD [2], being the most well-known underlying mechanism of cognitive decline. Apart from amyloidosis, white matter hyperintensities (WMH) have been associated with cognitive decline [3] and dementia [4,5]. Recently, the previous hypothesis that WMH were solely related to the vascular aspect of dementia [6] has been considered insufficient to explain the intricate relationship between WMH and dementia [7], as non-vascular mechanisms might be involved as well.

The consensus today is that the development of dementia is determined by both environmental and genetic factors. The most common type of dementia, AD, is considered a polygenic disease [8], as the presence of multiple polymorphisms in specific proteins, such as the apolipoprotein E (*ApoE*) [9], might increase the likelihood of developing the disease and, thus, dementia. In fact, the heritability of AD is high ranging, from 60% to 80% [10]. Relevant genome-wide association studies (GWAS) have revealed common single nucleotide polymorphisms (SNPs) associated with an increased risk of developing AD-type dementia [11] and have been used to calculate Polygenic Risk Scores (PRSs). Therefore, PRSs have been used as predictors of the risk for disease [12].

Apart from predictors of disease risk, relevant PRSs have also been investigated as possible indicators of cognitive decline [13]. An AD PRS might even exhibit a stronger relationship with cognitive decline compared to a PRS specific to cognitive function [14]. Existing studies have demonstrated distinct differences across specific cognitive domains. For instance, Gustavson et al. [15] and Darst et al. [16] have shown that the association of AD-PRSs and higher rates of cognitive decline (in memory and executive function) are driven by the effect of *APOE*. Xu et al. [17] have concluded that the predictive capacity of an AD-PRS in cognitive decline is independent of age and *APOE* only in the domain of executive function. In contrast, other studies [18,19] have highlighted that AD-PRSs are not able to predict cognitive decline over time in an aging population, asserting that these are solely associated with baseline cognitive ability. Thus, the existing literature presents conflicting data concerning the association of PRS related to dementia and longitudinal cognitive decline.

At the same time, data regarding the possible relationship between genetic propensity for specific pathways related to dementia pathophysiology and global or domain-specific cognitive decline are quite limited. From the aforementioned studies, only two [16,17] have explored PRSs related to specific pathways in the context of A_β_ metabolism, endocytosis, Tau pathology, and immune responses without promising results. In fact, a PRS for A_β_ metabolism was associated with a decline solely in the delayed recall score after excluding *APOE* [16], while all relationships in the other study were driven by the effect of *APOE* [17].

Therefore, we aimed to fill that literature gap by investigating the possible correlation of two PRSs, which are specific to distinct aspects of dementia pathophysiology (PRS Aβ_42_ and PRS WMH) and the rate of cognitive decline in cognitively normal (CN) older adults, as well as whether such an association might be influenced by age, sex, and cognitive reserve (CR), as proxied by the number of years of formal education. Our hypothesis was that increased risk for amyloidosis and WMH might be related to global and domain-specific cognitive decline in a population-based sample of older adults.

## 2. Results

### 2.1. Baseline Clinical and Socio-Demographic Characteristics

In total, 512 participants in the Hellenic Longitudinal Investigation of Aging and Diet (HELIAD) study, along with the available genomic data, were included in our analyses. The participants were followed longitudinally over time, with a mean follow-up of 2.9 years.

The baseline clinical and socio-demographic characteristics of all participants divided into classes of low/high PRS Aβ_42_ and PRS WMH can be found in Table 1. Those who had a higher PRS Aβ_42_ were older (*p* = 0.03) and had a greater global cognition (GC) score (*p* = 0.042) compared to those in the low PRS Aβ_42_ group. Clinical and demographic characteristics at baseline did not differ between PRS WMH groups.

The comparison of the clinical and socio-demographic characteristics when classifying participants according to global cognitive (GC) performance can be found in Table 2. Individuals with higher GC scores were younger and had more education years (*p* < 0.001) in comparison to participants belonging to the low GC group.

### 2.2. PRSs and Cognitive Decline

**Figure 1:**
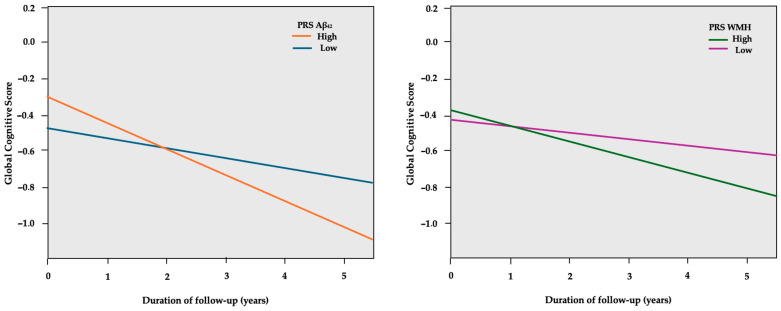
GEE predicted global cognitive scores (*y*-axis) over the course of follow-up in years (*x*-axis), separately for the low and high PRS Aβ_42_ groups. The model is adjusted for age, sex, education years, PC1, PC2, and *APOE* genotype.

Compared to the low PRS groups, the high PRS Aβ_42_ group was associated with a 4.2% higher standard deviation (SD) decline per year in the global composite score (Table 3), while the high PRS WMH group was related to a 2.9% higher SD decline per year, as presented in Figure 1. The results regarding individual cognitive domains were not significant. Analyses were adjusted for age, sex, CR, and *APOE* genotype, as well as the first two principal components (PC1, PC2) of genetic ancestry.

**Figure 2:**
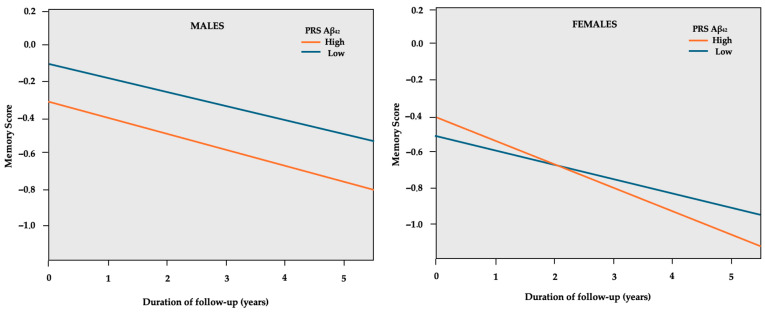
GEE predicted memory z-scores (*y*-axis) over the course of follow-up in years (*x*-axis) separately for the low and high PRS Aβ_42_ in sex subgroups. The model is adjusted for age, education years, PC1, PC2, and *APOE* genotype.

In sex-stratified GEE models, higher PRS groups in both males and females were associated with a decline in global cognition (Table 4). However, higher PRSs were related to significant memory decline only in females. A higher PRS Aβ_42_ was related to a 3.8% higher SD decline per year, as shown in Figure 2, while higher PRS WMH was related to a 3.0% higher SD decline in memory per year.

After age stratification, higher PRS groups in both younger and older participants were associated with a decline in global cognition (Table 5). PRSs were related to significant memory decline only in the older age group (higher PRS Aβ_42_ was related to a 3.9% higher SD decline per year and higher PRS WMH to 2.7%, respectively).

**Figure 3:**
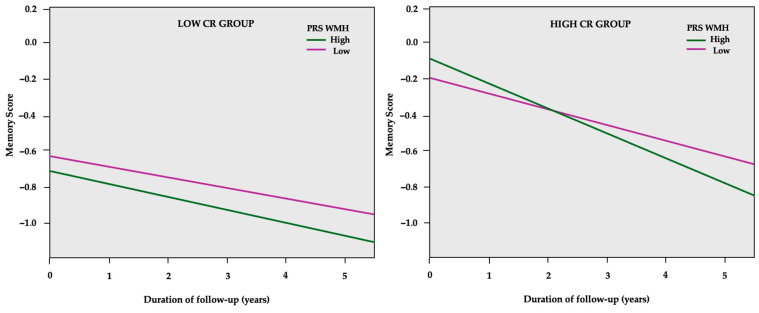
GEE predicted memory z-scores (*y*-axis) over the course of follow-up in years (*x*-axis) separately for the low and high PRS WMH in cognitive reserve (CR) subgroups. The model is adjusted for age, sex, PC1, PC2, and *APOE* genotype.

In CR-stratified models, a decline in global cognition was associated with higher PRS groups regardless of CR status (Table 6). Concerning the specific cognitive domains, PRSs were related to significant memory decline only in the high CR group (consisting of individuals with over 6 years of formal education). Specifically, a higher PRS Aβ_42_ was related to a 4.6% higher SD memory decline, while higher PRS WMH led to a 3.2% higher SD memory decline, as shown in Figure 3.

All the aforementioned *p*-values were corrected using the Benjamini–Hochberg procedure for multiple testing correction (as described in Section 4.5).

## 3. Discussion

In this population-based study, including individuals over 64 years of age, we investigated the predictive capacity of PRS Aβ_42_ and PRS WMH on the rate of cognitive decline over a 2.9-year (on average) follow-up period. We observed a relationship between higher genetic predisposition (as indicated by PRSs) for Aβ accumulation and WMH burden and an increased rate of global cognitive decline over time among individuals who were cognitively normal at baseline. Notably, in the whole study sample, we did not detect a statistically significant association with any of the cognitive subdomains assessed.

In stratified analyses, high PRS Aβ_42_ and high PRS WMH remained significantly associated with faster global cognitive decline independently of sex, age, or CR. Furthermore, we found an association between high PRSs and faster memory decline, which was detectable only among women, older individuals, and high-CR participants. Thus, stratification analysis has provided age, sex, and CR-dependent differences in memory function.

To date, and to our knowledge, no previous study has investigated the possible association of a PRS, which is specific for A_β_ accumulation or WMH burden and longitudinal cognitive change, either in terms of global cognition or specific cognitive domains. The majority of studies have only used PRSs specific for AD, which were constructed using SNPs associated with late-onset AD derived from relevant GWAS. An AD PRS has been found to be related to longitudinal cognitive decline (global or domain-specific), independently of the *APOE* genotype, in six studies [16,20,21,22,23,24]. The associations observed concern mainly the subdomains of memory and executive function. In contrast, other studies have not demonstrated any significant association between a PRS specific for AD and cognitive decline [18,19,25], or the observed association was driven by the influence of the *APOE* genotype [14,15,26]. Ritchie et al. [18] further elaborated that AD PRS was associated only with the baseline cognitive status but not the rate of cognitive decline.

Only two of the aforementioned studies have used PRSs specific for pathways related to dementia, such as PRSs for Aβ clearance and metabolism, which are conceptually closer to our study. In particular, Xu et al. [16] have shown that a PRS related to Aβ metabolism was associated with steeper memory (delayed recall) decline, an association which was controlled for *APOE*. In contrast, Darst et al. [15] concluded that the association between a pathway-specific PRS for Aβ clearance (consisting of 21 relevant SNPs apart from *APOE*) and cognitive decline was driven by the inclusion of *APOE* in the specific PRS. Furthermore, genetic predisposition to a higher WMH burden expressed using PRSs has been related to AD incidence in a limited number of studies [23,27], with no study investigating a possible relationship with the cognitive decline rate. The observed inconsistencies may stem from variations in sample characteristics among different studies as well as the inclusion of different SNPs in relevant GWASs in order to estimate genetic predisposition and calculate relevant PRSs for dementia. In fact, our population is South European, while most studies have included central and north European populations (in the UK and Sweden) with probable differences in genetic architecture.

The memory subdomain has been consistently associated with aging [28], while sex differences in memory performance have not been thoroughly investigated. In our study, elevated PRSs were significantly linked to memory decline only in females. Existing evidence suggests that women exhibit stronger memory skills compared to men, a trend that appears to remain consistent throughout the lifespan [29,30,31]. Some studies have shown a more pronounced memory decline in women during their eighth decade [32,33,34], which could be a possible explanation for our findings, as in our study sample, the mean age was 73.4 years. Moreover, women with an existing cognitive impairment have been shown to progress more quickly to MCI and dementia than men [35,36]. Thus, our findings are in accordance with the above assumption, as higher genetic predisposition for both Aβ_42_ and WMH are risk factors for dementia development. Sex-based variations in genetic risk between males and females have also been observed [37], as, for instance, women carrying two *APOE* ɛ4 alleles have been shown to demonstrate poorer memory performance between the ages of 65 and 69 compared to their male counterparts with the same genotype [38,39]. However, these findings have not yet been extended in the context of polygenic risk. In any case, opposing studies propose that males and females experience similar rates of cognitive decline [40]. Thus, further investigation is required to clarify the decline of sex differences in memory within the context of dementia.

Interestingly, as far as CR is concerned, we observed that individuals possessing a higher CR exhibited a more pronounced memory decline. In general, CR, as depicted by education years, is considered a protective factor for dementia development, as the vast majority of existing literature acknowledges its protective effect, highlighting a slower rate of memory decline in persons with high CR [41,42]. However, several studies are in accordance with our findings, showing a more rapid memory decline in patients with higher educational attainment [43,44,45], while a few studies have reported no difference in the rate of cognitive decline [46,47]. Our findings may indicate that the mechanism by which higher CR affects cognitive function in the dementia continuum is by delaying the onset of symptoms rather than reducing the rate of cognitive decline.

Our study presents some limitations. As real-time measurements of Aβ_42_ from CSF or PET scans were not available, as well as MRI scans, we were not able to assess the actual predictive capacity of PRSs for amyloid accumulation and the presence of WMHs. Moreover, the assessment of genetic predisposition through PRSs was based solely on common variants identified in GWASs, thus overlooking other biological factors known to influence or predict Aβ_42_ and WMH burden (rare variants, haplotypes, and epigenetic elements). Additionally, the duration of follow-up for this study was relatively short (2.9 years) in relation to the evolution of dementia, which occurs over a longer timeframe. Last but not least, the average educational level of our cohort was 7.1 years, which may restrict the generalizability of our results.

The present study also has several strengths. Firstly, we are unaware of another study investigating the effects of high genetic propensity for Aβ_42_ and WMH on the cognitive decline rate longitudinally. Moreover, the PRS approach offers several advantages, as it reduces potential errors associated with different methods of measuring CSF Aβ_42_ across different centers, as well as the concerns related to PET and MRI scans in terms of procedure and cost (e.g., selection of the Aβ_42_-PET positive threshold, especially in cases of low amyloid accumulation). Furthermore, the neuropsychological testing included thorough assessments for specific subdomains of cognition conducted by experts through detailed interviews.

## 4. Materials and Methods

### 4.1. Participants and Procedures

The study participants were derived from the HELIAD study, which is an epidemiological study of aging in the elderly Greek population focusing on dementia and other neuropsychiatric disorders. The recruitment process involved the random selection of individuals over 64 years old from an Athens suburb (Marousi) and the city of Larissa, as well as its rural surroundings, using local municipality registries.

Overall, 1986 individuals completed the baseline evaluation from 2011 to 2015, while 1226 participants completed the follow-up evaluation from 2013 to 2019. For our analyses, we only included HELIAD participants who (i) had available cognitive follow-up data, (ii) were not genetically related, (iii) did not have a baseline diagnosis of dementia or amnestic mild cognitive impairment (aMCI), and (iv) had available genotypic data (concerning the *APOE* genotype as well as PRS Aβ_42_ and PRS WMH). The final analytic sample consisted of 512 individuals.

All participants had provided informed consent before taking part in the study. All study procedures were approved by the Institutional Ethics Review Boards of the University of Thessaly and the National and Kapodistrian University of Athens. Information concerning medical and family history, lifestyle, and demographics (including age, sex, and education years) were collected during face-to-face interviews from the participants or their caregivers (first-degree relatives or spouses) when needed. Details about the design and key features of the HELIAD study and data collection procedures have been previously provided [48,49].

### 4.2. Neuropsychological Assessment

In both HELIAD visits (baseline and first follow-up) a thorough and detailed neuropsychological assessment was performed by trained neuropsychologists. A neuropsychological test battery, which is described in detail in the Supplementary Materials (Section S1.1), was used to evaluate global cognition and specific cognitive domains including memory, executive function, visuospatial ability, language, and attention/processing speed.

Each cognitive test score was converted to a z-score, using the mean and standard deviation values from the subset of participants without dementia or mild cognitive impairment (MCI) diagnosis. The z-scores of all tests within a specific domain were first averaged and then normalized again by their mean and standard deviation (SD) to create a domain-specific score. Finally, a z-score for global cognition was obtained by normalization of the averaged domain z-scores, with higher scores indicating better cognitive performance.

### 4.3. Genotyping and Imputation

Genome-wide genotyping was conducted using the Illumina Infinium Global Screening Array at the Life & Brain facilities in Bonn, Germany, the ‘Centre National de Recherche en Génétique Humaine’ (CNRGH) in Evry, France) and the Erasmus Medical Center in Rotterdam, Netherlands [12] as part of the European Alzheimer & Dementia Biobank (EADB) project. Calling was generated by the CNRGH in Evry, France, using the data generated by all centers involved in genotyping. Detailed information regarding the genotyping and imputation in the HELIAD study have been provided in a previously published work [50,51] and can be found in the Supplementary Materials (Section S1.2).

### 4.4. Polygenic Risk Score Estimation

Genetic predisposition for amyloid accumulation was modeled through a PRS, constructed by aggregating the effects of common genetic variants associated with cerebrospinal fluid (CSF) levels of Aβ_42_ [52]. In particular, the PRS Aβ_42_ was developed based on the summary statistics of a GWAS for CSF Aβ_42_ [24], in which each single nucleotide polymorphism (SNP) was associated with CSF Aβ_42_ levels at a certain *p*-value threshold. For each participant, we computed different PRSs for CSF Aβ_42_ based on a prior set of 10 *p*-value GWAS thresholds (P_T_) (i.e., 5 × 10^−5^, 0.0001, 0.001, 0.05, 0.01, 0.1, 0.2, 0.3, 0.4, 0.5). Given that Aβ_42_ levels in CSF are inversely related to the accumulation of Aβ_42_ in the brain, PRS values were multiplied by –1 to align a higher score with a higher genetic predisposition for increased Aβ_42_ levels in the brain.

Similarly, genetic propensity for a higher WMH burden was assessed through a PRS calculated using data from a meta-analysis of GWASs related to WMH volume, which was conducted by the CHARGE consortium [53]. The meta-analysis incorporated summary statistics from 23 population-based studies (n = 24,182), encompassing a total of 21,666 European individuals. Individuals with a history of stroke, brain tumors, or head trauma, as well as brain infarctions impacting the gray matter identified through MRI, were excluded. The methodology we followed for the calculation of the PRSs can be found in the Supplementary Materials (Section S1.3).

### 4.5. Statistical Analysis

The statistical analyses were performed using SPSS 28.0. Participant characteristics were expressed as mean values ± SD for continuous variables or as percentages for categorical variables. To compare the baseline socio-demographic characteristics of participants, we ranked the baseline PRSs and global cognitive scores into two equal groups (low-high) based on the median values for each variable. These groups were compared using Pearson’s chi-squared test for categorical variables, while analysis of variance (ANOVA) was performed for the comparison of continuous variables. The level of significance was set at *p* < 0.05.

PRSs were treated as dichotomous variables, with the median used as a cut-off (0: low PRS and 1: high PRS). The values of the medians were 0.028 for PRS Aβ_42_ and −0.025 for PRS WMH, respectively. Regarding stratified analyses, we performed sex (males vs. females), age (younger vs. older group using the median of 72.68 years as cut-off), and CR stratification (using 6 education years as cut-off, which corresponds to the cut-off for primary/elementary education in Greece).

For our investigation, we specifically chose the P_T_s that exhibited the highest accuracy in classifying AD/aMCI cases versus non-AD/aMCI cases in our cohort, as done in previous studies [50,51,54]. Therefore, we chose the p_T_ < 0.1 for PRS Aβ_42_ and p_T_ < 0.3 for PRS WMH (as shown in Section S1.4, Tables S1 and S2 of the Supplementary Materials).

We used generalized estimating equations (GEE) models to examine whether PRSs might be associated with differential rates of cognitive change over time. The reason why we chose the specific models is the fact that GEE models extend the generalized linear model, allowing for analysis of repeated measurements (thus, the relationship between the predictor and the outcome remains linear). In particular, GEE models take into consideration the multiple visits per individual, as well as the fact that the characteristics of the same individual subjected to repeated measurements over time might be correlated [55]. We treated each participant’s baseline and follow-up evaluations as a cluster.

We constructed six consecutive GEE models for each PRS. GEE analyses featured the main effects of each PRS (as a dichotomous variable) and time from baseline, as well as PRS by time interaction terms. Specifically, PRS Aβ_42_/PRS WMH, time (follow-up duration in years from baseline assessment), as well as PRS x follow-up duration interaction were the main predictors in independent models. The global and domain-specific cognitive z-scores (memory, executive function, visuospatial ability, language, and attention) were used as the dependent scale variables. A significant interaction term would indicate differential rates of cognitive change as a function of baseline PRS.

Therefore, the value of ‘β’, which is the regression coefficient of GEE models, corresponds to the difference in the cognitive change rate between the high PRS and the low PRS group (as PRSs were treated as dichotomous variables, using the low PRS group as reference) per year of follow-up. The difference is expressed as a percentage of one unit of SD of the relevant cognitive score. All analyses were adjusted for age, sex, CR, and *APOE* genotype, as well as the first two principal components (PC1, PC2) of genetic ancestry.

To explore potential disparities regarding the impact of PRS on different sexes, ages, and CRs, we performed subgroup analyses using the same approach described above. Given that we examined multiple PRSs and multiple cognitive domains, we performed multiple testing corrections. We have corrected *p*-values using the Benjamini–Hochberg procedure [56]. The false discovery rate (FDR) was controlled at <5%.

## 5. Conclusions

In conclusion, in our study, two PRSs related to different aspects of dementia pathophysiology (i.e., amyloid deposition and WMHs) independently predicted a more rapid rate of global cognitive decline in a sample of adults older than 64 years old. Therefore, the specific genetic predictors might be used to recognize individuals with an increased genetic risk of cognitive decline before the onset of clinical symptoms, who may be promising candidates for clinical trials concerning new dementia treatments. Moreover, the two PRSs were also associated with memory decline; nevertheless, the specific relationships were sex-, age-, and CR-dependent. Thus, these factors should be taken into account when investigating genetic predictors for dementia. In any case, further longitudinal studies are needed to validate the aforementioned genetic predictors, elucidate the impact of genetic predisposition on pathways related to dementia pathophysiology and cognitive decline, and clarify specific sex and CR differences.

## Figures and Tables

**Table 1:** Participants’ baseline characteristics by groups of polygenic risk scores.

	All Participants	PRS ^1^ Aβ_42_			PRS WMH		
		Low	High		Low	High	
	N = 512	N = 256	N = 256	*p*-Value	N = 256	N = 256	*p*-Value
Age (years), mean ± SD ^2^	73.4 ± 4.9	72.8 ± 4.5	74.0 ± 5.3	**0.030**	73.7 ± 5.1	73.2 ± 4.7	0.238
Sex, females (%)	290 (56.6)	148 (57.8)	142 (55.5)	0.467	135 (52.7)	155 (60.5)	0.084
Education years, mean ± SD	7.1 ± 4.5	7.0 ± 4.6	7.2 ± 4.4	0.662	7.2 ± 4.4	7.0 ± 4.5	0.623
Follow-up duration, mean ± SD	2.9 ± 0.8	3.0 ± 0.9	2.9 ± 0.8	0.156	2.9 ± 0.8	3.0 ± 0.8	0.167
*APOE* ε4 carriers, yes (%)	86 (16.8)	38 (14.8)	48 (18.8)	0.188	42 (16.4)	44 (17.2)	0.804
Global score, mean ± SD	−0.39 ± 0.79	−0.47 ± 0.86	−0.32 ± 0.71	**0.042**	−0.41 ± 0.85	−0.37 ± 0.74	0.530
Memory score, mean ± SD	−0.32 ± 0.90	−0.38 ± 0.93	−0.26 ± 0.87	0.124	−0.28 ± 0.94	−0.36 ± 0.86	0.325
Attention score, mean ± SD	−0.40 ± 1.21	−0.50 ± 1.37	−0.30 ± 1.02	0.082	−0.43 ± 1.26	−0.36 ± 1.17	0.459
Visuospatial score, mean ± SD	−0.42 ± 0.94	−0.47 ± 1.01	−0.36 ± 0.86	0.222	−0.45 ± 1.03	−0.38 ± 0.84	0.509
Executive score, mean ± SD	−0.34 ± 0.78	−0.30 ± 0.72	−0.38 ± 0.85	0.303	−0.36 ± 0.78	−0.32 ± 0.78	0.525
Language score, mean ± SD	−0.37 ± 0.89	−0.44 ± 0.92	−0.31 ± 0.85	0.105	−0.39 ± 0.93	−0.35 ± 0.85	0.550

^1^ Polygenic Risk Score, ^2^ Standard Deviation. Bold values indicate statistical significance.

**Table 2:** Participants’ baseline characteristics by global cognition at baseline.

	All Participants	Low GC ^1^ Group	High GC ^1^ Group	
	N = 512	N = 256	N = 256	*p*-Value
Age (years), mean ± SD ^2^	73.4 ± 4.9	75.1 ± 4.7	71.8 ± 4.5	**<0.001**
Sex, females (%)	290 (56.6)	141 (55.1)	149 (58.2)	0.585
Education years, mean ± SD	7.1 ± 4.5	4.9 ± 3.1	9.4 ± 4.5	**<0.001**
Follow-up duration, mean ± SD	2.9 ± 0.8	3.0 ± 0.9	2.8 ± 0.8	0.146
*APOE* ε4 carriers, yes (%)	86 (16.8)	47 (18.4)	39 (15.2)	0.397
PRS Aβ_42_, high (%)	256 (50.0)	133 (52.0)	123 (48.0)	0.416
PRS WMH, high (%)	256 (50.0)	131 (51.2)	125 (48.8)	0.641

^1^ Global Cognition, ^2^ Standard Deviation. Bold values indicate statistical significance.

**Table 3:** Results from independent adjusted GEE models concerning the association between baseline PRSs and differential rates of change of cognitive composite scores in the whole sample.

	Global		Memory		Executive		Visuospatial		Language		Attention	
N = 512	Β ^1^	*p*	β	*p*	β	*p*	β	*p*	β	*p*	β	*p*
PRS ^2^ Aβ_42_	−0.042	**0.002**	−0.025	0.198	−0.028	0.161	−0.005	0.464	−0.024	0.273	−0.020	0.375
PRS WMH	−0.029	**0.037**	−0.016	0.325	−0.017	0.146	−0.005	0.439	−0.013	0.468	−0.012	0.348

^1^ Regression Coefficient of GEE models, ^2^ Polygenic Risk Score. Bold values indicate statistical significance.

**Table 4:** Results from sex-stratified independent adjusted GEE models concerning the association between baseline PRSs and differential rates of change of cognitive composite scores.

Males	Global		Memory		Executive		Visuospatial		Language		Attention	
N = 222	β ^1^	*p*	β	*p*	β	*p*	β	*p*	β	*p*	β	*p*
PRS ^2^ Aβ_42_	−0.029	**0.019**	−0.012	0.337	−0.025	0.306	−0.008	0.309	−0.014	0.329	−0.022	0.278
PRS WMH	−0.028	**0.039**	−0.003	0.341	−0.010	0.351	−0.010	0.309	0.003	0.324	−0.020	0.180
**Females**	**Global**		**Memory**		**Executive**		**Visuospatial**		**Language**		**Attention**	
**N = 290**	**β**	***p***	**β**	***p***	**β**	***p***	**β**	***p***	**β**	***p***	**β**	***p***
PRS Aβ_42_	−0.057	**<0.001**	−0.038	**0.012**	−0.029	0.252	−0.003	0.335	−0.031	0.091	−0.018	0.103
PRS WMH	−0.031	**0.023**	−0.030	**0.031**	−0.025	0.260	−0.003	0.342	−0.029	0.095	−0.004	0.347

^1^ Regression Coefficient of GEE models, ^2^ Polygenic Risk Score. Bold values indicate statistical significance.

**Table 5:** Results from age-stratified independent adjusted GEE models concerning the association between baseline PRSs and differential rates of change of cognitive composite scores.

Younger Group	Global		Memory		Executive		Visuospatial		Language		Attention	
N = 256	β ^1^	*p*	β	*p*	β	*p*	β	*p*	β	*p*	β	*p*
PRS ^2^ Aβ_42_	−0.036	**0.013**	−0.011	0.299	−0.026	0.305	−0.011	0.302	−0.025	0.112	−0.019	0.298
PRS WMH	−0.027	**0.040**	−0.007	0.302	−0.010	0.304	−0.010	0.301	−0.024	0.097	−0.011	0.314
**Older Group**	**Global**		**Memory**		**Executive**		**Visuospatial**		**Language**		**Attention**	
**N = 256**	**β**	***p***	**β**	***p***	**β**	***p***	**β**	***p***	**β**	***p***	**β**	***p***
PRS Aβ_42_	−0.048	**0.002**	−0.039	**0.002**	−0.029	0.214	−0.002	0.425	−0.023	0.152	−0.021	0.276
PRS WMH	−0.030	**0.021**	−0.027	**0.005**	−0.023	0.219	−0.002	0.473	−0.003	0.356	−0.014	0.340

^1^ Regression Coefficient of GEE models, ^2^ Polygenic Risk Score. Bold values indicate statistical significance.

**Table 6:** Results from independent CR-stratified adjusted GEE models concerning the association between baseline PRSs and differential rates of change of cognitive composite scores.

Low CR ^1^	Global		Memory		Executive		Visuospatial		Language		Attention	
N = 340	β ^2^	*p*	β	*p*	β	*p*	β	*p*	β	*p*	β	*p*
PRS ^3^ Aβ_42_	−0.047	**<0.001**	−0.006	0.301	−0.032	0.145	−0.008	0.366	−0.030	0.073	−0.023	0.125
PRS WMH	−0.029	**0.031**	−0.008	0.300	−0.016	0.302	−0.010	0.345	−0.007	0.386	−0.013	0.314
**High CR**	**Global**		**Memory**		**Executive**		**Visuospatial**		**Language**		**Attention**	
**N = 172**	**β**	***p***	**β**	***p***	**β**	***p***	**β**	***p***	**β**	***p***	**β**	***p***
PRS Aβ_42_	−0.039	**0.004**	−0.046	**<0.001**	−0.023	0.247	−0.003	0.472	−0.022	0.123	−0.015	0.237
PRS WMH	−0.030	**0.021**	−0.032	**0.020**	−0.019	0.272	−0.003	0.415	−0.004	0.401	−0.010	0.407

^1^ Cognitive Reserve, ^2^ Regression Coefficient of GEE models, ^3^ Polygenic Risk Score. Bold values indicate statistical significance.

## Data Availability

The data that support the findings of this study are available from the study’s principal investigator, N.S., upon reasonable request.

## Supplementary Materials

The following supporting information can be downloaded at: https://www.mdpi.com/article/10.3390/ijms26030910/s1, S1.1: Neuropsychological Evaluation [57,58,59,60,61,62,63,64,65]; S1.2: Genotype Imputation in HELIAD [66,67,68,69,70,71]; S1.3: Polygenic Risk Score Calculation; S1.4: Polygenic Risk Score Thresholds [72,73]; Table S1: Number of SNPs included at each PRS Aβ42 calculated at different GWAS *p*-value thresholds. AUC area together with *p* value of each PRS derived from a logistic regression with outcome aMCI/AD status, adjusted for *APOE* e4 genotype, PC1 and PC2. Table S2: Number of SNPs included at each PRS WMH calculated at different GWAS *p*-value thresholds. AUC area together with *p* value of each PRS derived from a logistic regression with outcome aMCI/AD status, adjusted for *APOE* e4 genotype, PC1 and PC2.
